# A case of giant ileal duplication in an adult, successfully treated with laparoscope-assisted surgery

**DOI:** 10.1186/s40792-015-0019-3

**Published:** 2015-01-30

**Authors:** Yasunori Matsumoto, Takayuki Tohma, Hideaki Miyauchi, Kazufumi Suzuki, Takanori Nishimori, Gaku Ohira, Kazuo Narushima, Yorihiko Muto, Tetsuro Maruyama, Hisahiro Matsubara

**Affiliations:** Department of Frontier Surgery, Graduate School of Medicine, Chiba University, 1-8-1Inohana, Chuo-Ku, Chiba-Shi, Chiba 260-8670 Japan

**Keywords:** Ileal duplication, Giant, Laparoscope, Adult

## Abstract

Alimentary tract duplication is a rare congenital malformation but can occur anywhere along the digestive tract. Most patients become symptomatic in early childhood, and only a few cases of adult patients have been reported in the literature. We herein report a unique case of a giant ileal duplication in an adult, which was successfully treated with laparoscope-assisted surgery. A 60-year-old male was admitted because of abdominal pain. Imaging studies revealed a well-defined cystic mass, measuring 15 cm, in the ileocecal region. We diagnosed it as a duplicated ileum and performed laparoscope-assisted surgery. The duplication was successfully resected with attached normal ileum, and there were no major complications in the postoperative course.

## Background

Duplication of the alimentary tract is a rare congenital malformation that can arise throughout the alimentary tract from the oral cavity to the anus [1]. Most patients are diagnosed in their infancy or childhood. More than 80% of cases present before they are 2 years old as an acute abdomen or bowel obstruction, while a minority may remain asymptomatic until adulthood [2]. We herein report a rare case of a giant ileal duplication in an adult, which was successfully treated with laparoscope-assisted surgery.

## Case presentation

A 60-year-old male was admitted to our hospital with a complaint of right lower abdominal pain. The patient did not have same symptoms before, and he only had a medical history of asthma. The laboratory data on admission showed a slightly elevated WBC count of 9,300/μl and a CRP level of 1.0 mg/dl, but the tumor marker levels (CEA, CA19-9) were almost within the normal limits. Abdominal computed tomography (CT) demonstrated a 15-cm large cystic mass, like a dilated intestine, and fluid collection was observed inside.

**Figure 1:**
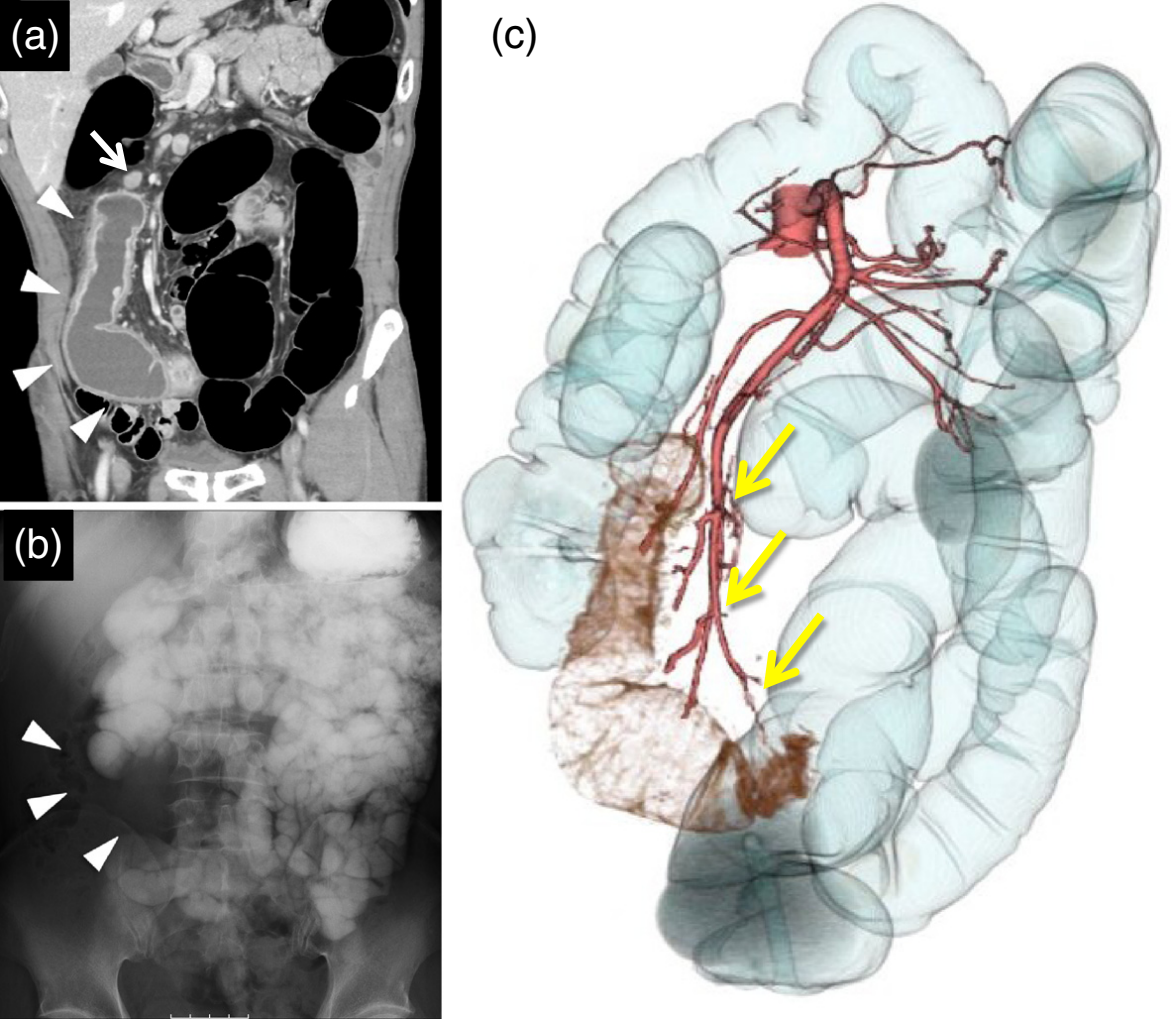
**Coronal reconstruction image, small bowel follow-through exam, and 3d reconstruction image. (a)** A coronal reconstruction image of a CT scan showed a tortuous tubular cyst that was 15 cm in diameter (arrowhead), and swelling of the lymph nodes was detected around the cyst (arrow). **(b)** A small bowel follow-through exam showed that the cyst did not come out, but that it excluded normal intestine (arrowhead). **(c)** A 3D reconstruction image showed that the feeding arteries were separated from the upper mesentric artery (arrow).

The cystic mass was adhered to the ileum, but there was no definite communication to the ileum. The feeding arteries of the cyst were communicating branches from the upper mesentery artery, and the lymph nodes around the cyst were slightly swollen (Figure 1a,c). At the small-bowel follow-through exam, the cyst did not come out, but it did exclude normal intestine (Figure 1b). There were not significant findings during a colonoscopic examination.

Based on these findings, we diagnosed the cyst as an ileal duplication and decided to perform surgery in order to prevent a recurrence of abdominal pain and lethal complications, such as bleeding or perforation.

**Figure 2:**
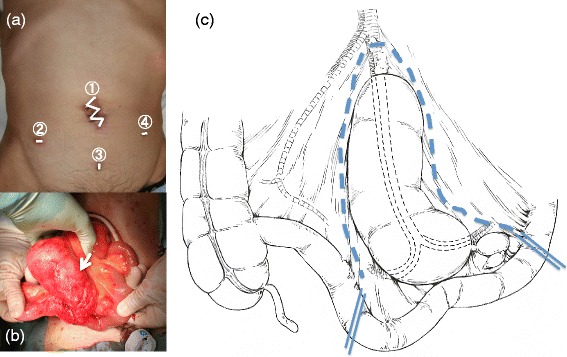
**Design of the skin incisions, intraoperative photograph, and schematic diagram. (a)** The design of the skin incisions. Laparoscope-assisted surgery was performed with four port, and the umbilical port was used for minilaparotomy with a zigzag skin incision. **(b)** An intraoperative photograph. The ileal duplication (arrow) was deforming the mesenteric side of the wall of the distal ileum. **(c)** A schematic diagram of the intraoperative findings. The exicision line is shown with a blue dotted line.

We employed a four-port laparoscope-assisted resection of the duplicated ileum and segmental resection of the normal ileum. An umbilical port was used for a mini-laparotomy with a zigzag skin incision (Figure 2a). The cyst’s stalk was attached to the base of the mesentery of the terminal ileum, approximately 40 cm from the ileocecal junction (Figure 2b,c). After resection of the cyst, a side-to-side anastomosis of the ileum was made with a stapler.

**Figure 3:**
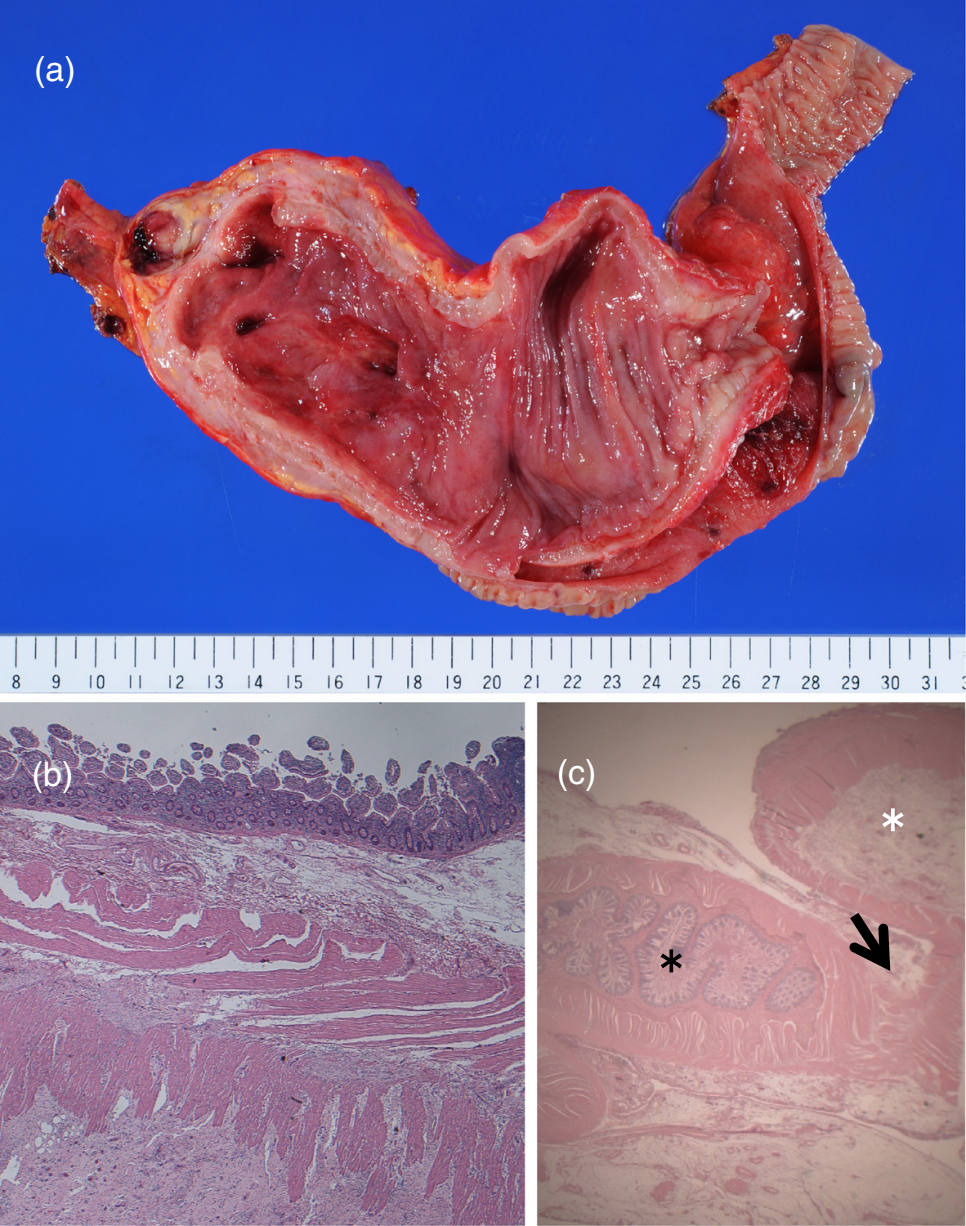
**Macroscopic findings, result of histological examination, and duplicated ileum. (a)** The macroscopic findings of the resected specimen. **(b)** The result of a histological examination of the duplication. The surface was covered with ileal mucosa and the duplication had muscle layers (H.E. stain × 100). **(c)**The duplicated ileum (white asterisk) shared the same muscle (arrow) as the adjacent ileum (black asterisk) (H.E. stain × 5).

The resected specimen showed an ileal duplication of 18 × 7 cm in diameter, filled with purulent matter with some ulcers inside (Figure 3a). The histological findings showed that it had a mucus gland inside, like intestinal epithelium, and was covered with two layers of smooth muscle. There was no communication between the duplication and normal ileum, but they had a common muscle layer (Figure 3b,c).

The postoperative period was uneventful without any asthma attack, and the patient was discharged on the 13th postoperative day.

## Discussion

Duplication of the alimentary tract is rare congenital anomaly observed in one out of 25,000 deliveries [3]. These anomalies are usually present in childhood and occasionally in adults, and can be encountered anywhere throughout the gastrointestinal tract, from the mouth to the anus. The terminal ileum is thought to be the most frequently involved [4].

Although symptoms such as vomiting, constipation, abdominal pain, obstruction, an abdominal mass, and hemorrhage have been reported, duplication can be easily misdiagnosed as Meckel’s diverticulum, appendicitis, a choledochal cyst, or Crohn’s disease, especially when it occurs in adults. Prior to surgery, it is difficult to diagnose alimentary duplication because of the non-specificity of symptoms and the presentation [5]. Indeed, only 11.2% of cases have been correctly diagnosed before surgery in Japan [6]. Recently, the usefulness of capsule endoscopy [7] or double balloon endoscopy [8] for the detection of the condition has been reported, but the disease still cannot be detected in patients who have no communication to the normal intestine.

Surgical treatment is advocated after a diagnosis of ileal duplication in order to prevent potentially lethal complications such as perforation, volvulus, intussusception, bowel obstruction, and enteric bleeding. Heterotopic mucosa of gastric or pancreatic origin are sometimes seen, with a frequency of 17%–36% for gastric mucosa [9], and these are thought to result in perforation or bleeding [10,11]. The duplication itself is also thought to have malignant potential [5,12,13]. In our case, the specimen had a similar physiological architecture to the small bowel, with indicated villi, crypts, and a two-layered muscular wall. The tissue of gastric or pancreatic origin was not observed.

Recently, some reports have suggested that laparoscope-assisted surgery could be useful for making a diagnosis and treating this condition [14,15]. Sixteen cases of alimentary tract duplication in adults, treated with laparoscopic surgery, have been reported in the Japanese literature [16-28]. Only four cases were diagnosed before surgery, but an accurate intraoperative diagnosis could be made in the rest of the cases, and accurate resection was performed. Our case had the largest cyst in the reported cases.

## Conclusions

In conclusion, it is difficult to diagnose alimentary duplications, and laparoscope-assisted surgery is thought to be beneficial for both making a diagnosis and performing accurate treatment.

## Consent

Written informed consent was obtained from the patient for publication of this case report and any accompanying images. A copy of the written consent is available for review by the Editor-in-Chief of this journal.
